# Photoluminescence enhancement in CdS quantum dots by thermal annealing

**DOI:** 10.1186/1556-276X-7-482

**Published:** 2012-08-29

**Authors:** Jae Ik Kim, Jongmin Kim, Junhee Lee, Dae-Ryong Jung, Hoechang Kim, Hongsik Choi, Sungjun Lee, Sujin Byun, Suji Kang, Byungwoo Park

**Affiliations:** 1 WCU Hybrid Materials Program, Department of Materials Science and Engineering, Research Institute of Advanced Materials, Seoul National University, Seoul 151-744, South Korea

**Keywords:** CdS quantum dot, photoluminescence, quantum efficiency, local strain, relaxation

## Abstract

The photoluminescence behavior of CdS quantum dots in initial growth stage was studied in connection with an annealing process. Compared to the as-synthesized CdS quantum dots (quantum efficiency ≅ 1%), the heat-treated sample showed enhanced luminescence properties (quantum efficiency ≅ 29%) with a narrow band-edge emission. The simple annealing process diminished the accumulated defect states within the nanoparticles and thereby reduced the nonradiative recombination, which was confirmed by diffraction, absorption, and time-resolved photoluminescence. Consequently, the highly luminescent and defect-free nanoparticles were obtained by a facile and straightforward process.

## Background

Due to the benefits of their size-tunable physical properties [1-3], nanoscale semiconductor materials have promising future applications, including the optoelectronic devices such as light-emitting diodes [4-8] and next-generation quantum dot solar cells [9-14]. Moreover, nanoscale semiconductors functionalized with biomolecules are used as molecular fluorescent probes in biological applications [15].

In recent years, there has been a rapid development of the growth techniques for quantum dots with high crystallinity and narrow size distribution [16-18]. The hot-injection techniques allow the affordable growth of a wide range of nanoscale materials with high quality [19-21]. On the other hand, low-temperature synthesis has not been actively studied yet. Low-temperature synthesis has higher potential than hot-injection techniques because the process is relatively simple and nontoxic [22]. However, the size distribution and the crystallinity of nanoparticles are generally poor because of low synthetic temperature and surface defects [23]. Recently, several papers have introduced advanced low-temperature synthesis and colloidal growth that can yield quantum dots with a sufficiently narrow size distribution [24-27].

In this regard, introducing a facile annealing process has great potential for enhancing the quantum efficiency and tuning the size of nanocrystals. However, systematic analysis of the initial growth stage of the nanoparticles has rarely been studied. In this work, a simple aqueous system and straightforward annealing process were applied to the preparation of highly luminescent CdS quantum dots. The appropriate annealing condition was well correlated with the quantum dot size, local strain (crystallinity), and radiative/nonradiative recombination rates.

## Methods

The CdS quantum dots were synthesized by using a combination of the reverse-micelle method and post-growth annealing process. Cadmium chloride (CdCl_2_, 0.182 g) and sodium sulfide (Na_2_S, 0.036 g) were separately dissolved in distilled water (15 ml) and stirred to achieve their complete dissolution. Linoleic acid ((C_17_H_31_)COOH, 2.4 ml) and sodium linoleate ((C_17_H_31_)COONa, 2 g) were dissolved in ethanol (15 ml) and formed transparent solutions. After the two solutions were mixed and stirred vigorously, the color changed from transparent to opaque white, implying the formation of a microemulsion consisting of cadmium linoleate. After the addition of sodium sulfide, the color changed from white to greenish yellow. For the annealing process, the autoclave was heated at 100°C for 1 to 24 h. In order to increase the quantum dot size, post-growth annealing was also conducted at 125°C to 225°C with the same annealing time (12 h). The resultant CdS quantum dots were precipitated by using centrifugation and cleaned several times with ethanol. Finally, the CdS quantum dots were dispersed into chloroform (CHCl_3_, 40 ml), displaying a translucent yellow solution.

The structural properties of the quantum dots, such as crystal size and local strain, were studied using X-ray diffraction (XRD; M18XHF-SRA, MAC Science, Yokohama, Japan) with *θ* to 2*θ* curves. To analyze the optical properties, the absorbance was measured using UV/visible spectrometry, and the photoluminescence (PL) data were measured under 360-nm excitation wavelength with a spectrofluorometer (FP-6500, JASCO, Essex, UK). The binding energy of CdS quantum dots was analyzed by X-ray photoelectron spectroscopy (XPS; Sigma Probe, Thermo VG Scientific, Logan, UT, USA) using Al *Kα* radiation (1,486.6 eV). Time-resolved PL was measured by using a picosecond laser system (FLS920P, Edinburgh Instruments Ltd., Livingston, UK), and the nanostructures of the CdS nanoparticles were analyzed by a high-resolution transmission electron microscopy (TEM; JEM-3000 F, JEOL Ltd., Tokyo, Japan).

## Results and discussion

**Figure 1:**
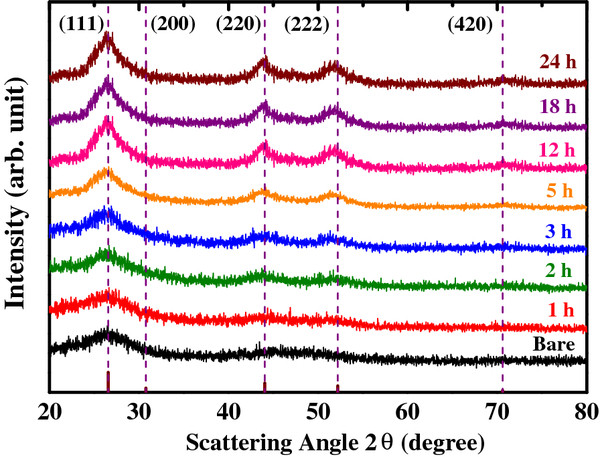
X-ray diffraction of CdS quantum dots with different annealing times at 100°C.

The effects of annealing on the size and crystallinity of CdS nanocrystals were investigated by XRD (Figure 1) with different annealing times. Due to the reaction of residual source during the annealing process plus coarsening behavior of nanoparticles, the average size of CdS quantum dot increases with the annealing time [24]. The diffraction peaks show the zinc-blende phase (JCPDS 75–0581) with no impurity phases. To estimate the nonuniform distribution of local strain (crystallinity) and grain size of CdS quantum dots, four diffraction peaks were fitted with the scattering vector $k=\left(4\pi /\lambda \right)\text{sin}\theta$ using a double-peak Lorentzian function, considering the effects of *Kα*_1_ and *Kα*_2 _[28-31] and the instrumental-broadening effect. Before the post-growth annealing process, the size of CdS quantum dots was estimated to be approximately 2.3 nm, which gradually grew to approximately 3.7 nm with increased annealing time (in the following Figure 6). As the annealing time increases, the local strain of CdS quantum dots decreases, suggesting that defects accumulated in the CdS quantum dots during the nucleation stage are relaxed by annealing.

**Figure 2:**
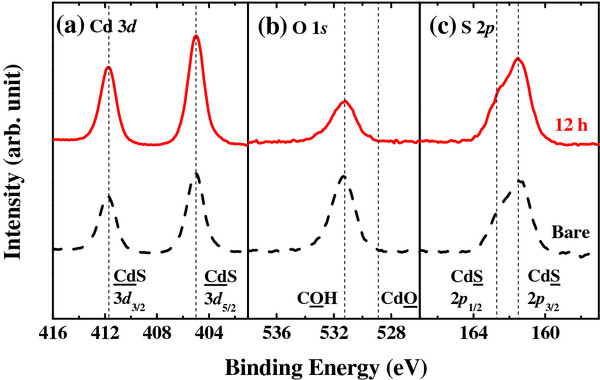
**XPS spectra.** (**a**) Cd 3*d*, (**b**) O 1*s*, and (**c**) S 2*p* with bare and 12-h-annealed samples at 100°C.

The change in the surface states of CdS quantum dots was examined by XPS (Figure 2). The XPS data of the O 1 *s* level display the chemical bonding of carboxyl acid, and the peak position of the Cd 3*d* level is not shifted after annealing. The dotted peak positions denote literature values [32-34]. If the sample becomes oxidized after heat treatment, the oxygen and cadmium peaks will display changes in the chemical bonding to cadmium sulfate [33-35]. This result revealed that the surface of the CdS quantum dots was not changed despite the heat treatment, which means that the linoleate surfactant is still bound to the nanocrystal surface after the 100°C annealing [26].

**Figure 3:**
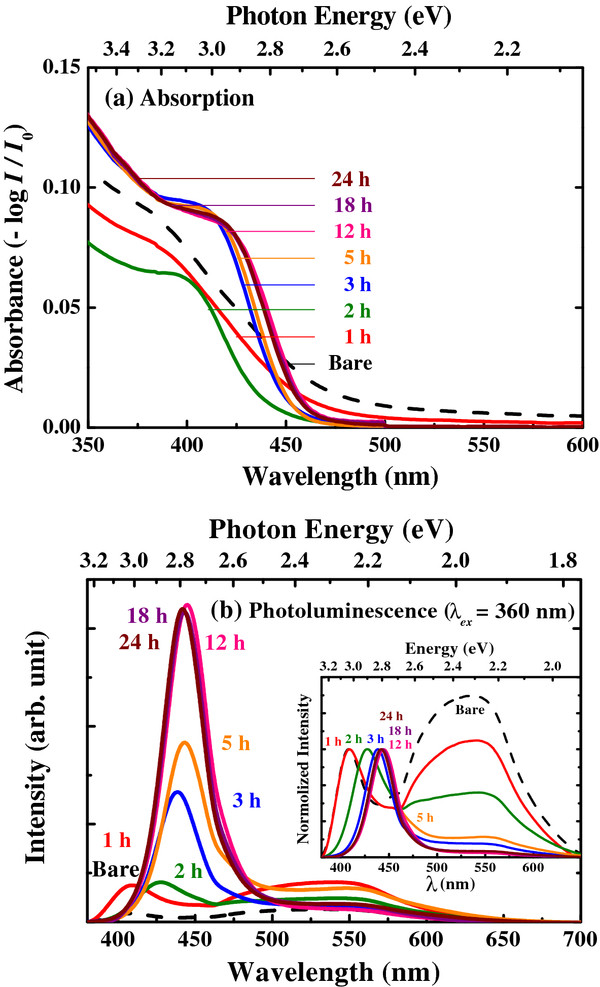
**Effects of annealing time at 100°C.** (**a**) Absorbance and (**b**) photoluminescence spectra of CdS nanoparticles as a function of annealing time at 100°C. The inset shows a normalized PL spectra.

Figure 3 shows several absorbance and PL spectra after different annealing times for the CdS nanoparticles. As the annealing time increases, the absorbance spectra exhibit a red shift relative to the bare sample (Figure 3a). The band-edge emission also shifts to higher wavelength because of the increase of quantum dot size during the annealing process (Figure 3b). The exciton peak becomes clear after 5 h of annealing, indicating improved crystallinity and size dispersity. It was observed that the absorbance intensity below the bandgap energy (*E*_*g*_) decreased with increasing annealing time, which suggests that the trap-state reduction has occurred in the CdS quantum dots [25]. For the bare sample (quantum efficiency ≅ 1%), broad emission ranging from 450 to 650 nm is dominant (Figure 3b), which originates from the trap-state emission [27,36]. In contrast, the CdS quantum dots annealed for 12 h exhibit strong and narrow band-edge emission at 440 nm (quantum efficiency ≅ 29%). These phenomena also strongly suggest that simple annealing at 100°C reduces the trap states in the CdS nanoparticles.

**Figure 4:**
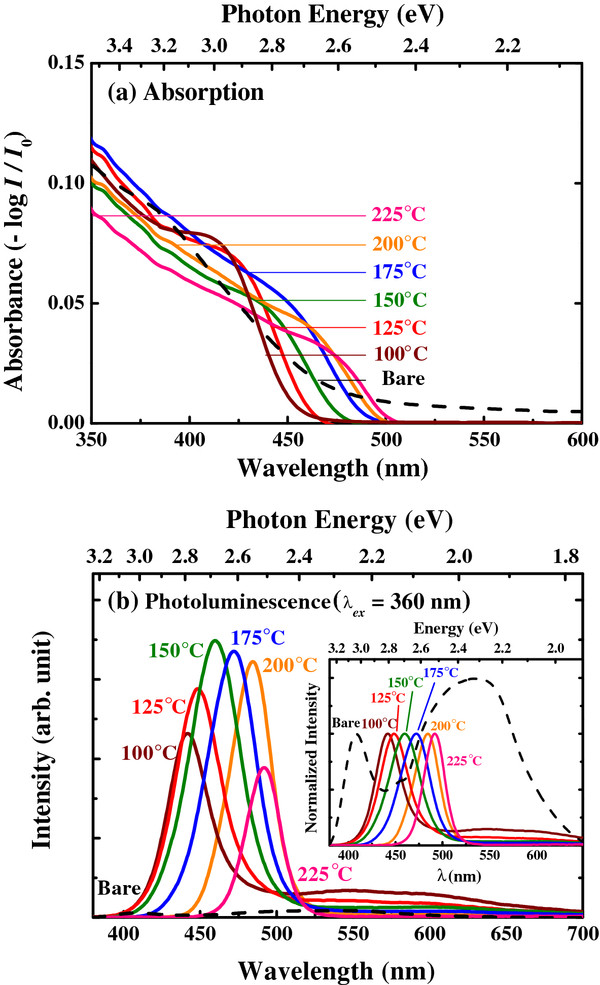
**Effects of annealing temperature for 12 h.** (**a**) Absorbance and (**b**) photoluminescence spectra of CdS nanoparticles as a function of annealing temperature for 12 h. The inset shows normalized PL spectra.

To control the radius of the nanoparticles, post-growth annealing process was further examined by varying the annealing temperature (125°C to 225°C). As shown in Figure 4a, the absorbance spectra show a red shift relative to the bare sample, confirming increased nanoparticle size with high annealing temperature. In addition, the wavelength of the band-edge emission was controlled from approximately 440 to approximately 490 nm by simply changing the annealing temperature, as shown in Figure 4b.

**Figure 5:**
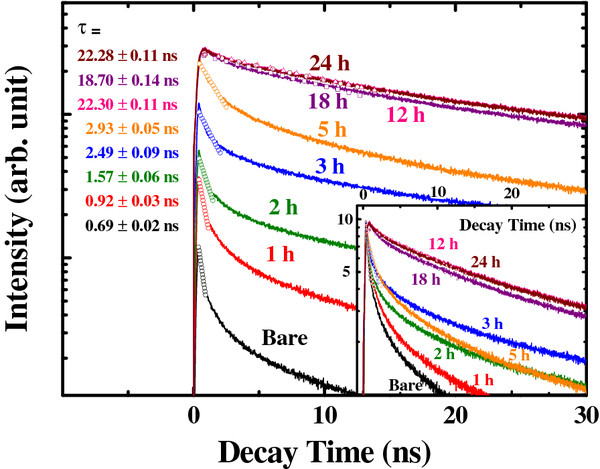
**Time-resolved PL data as a function of annealing time at 100°C.** The inset shows normalized data.

Time-resolved PL measurements were performed to determine the carrier dynamics of CdS quantum dots, as shown in Figure 5[37,38]. The bare sample exhibits fast initial relaxation. In contrast, the initial behavior of the post-annealed samples exhibits long life decay behavior. Even though the decay curves in Figure 5 do not show single-exponential behavior, the decay curves were fitted assuming single exponential in the initial stage because the initial decay occupies a large fraction of total recombination. The samples annealed over 12 h show nearly single-exponential decay, which indicates high crystallinity and the absence of defect-related decay channels [39,40]. This is consistent with the reduction of defect peak (approximately 550 nm) in Figure 3b after 12-h annealing. The quantum efficiency (*η*) is the ratio of radiative recombination to the total recombination as [41]:

(1) $$\eta =\frac{k_{\text{rad}}}{k_{\text{rad}}+k_{\text{nonrad}}}=k_{\text{rad}}\times \tau \text{,}$$

where *k*_total_*k*_rad_*k*_nonrad_, and *τ* are the total, radiative, nonradiative recombination rates, and decay time (*k*_rad_ + *k*_nonrad_)^−1^, respectively. The quantum efficiency of colloidal CdS samples was estimated by comparing with the emission of Rhodamine 6G in ethanol (quantum efficiency of approximately 95% for an excitation wavelength of 488 nm) [42]. Then, each decay time constant was obtained using the total recombination rates from the decay curves (Figure 5) and quantum efficiency.

**Figure 6:**
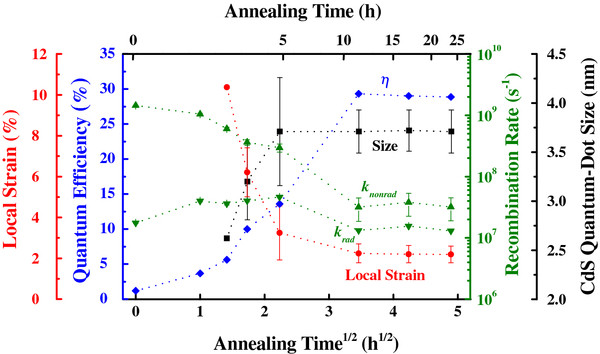
The correlation between local strain, quantum efficiency, recombination rate, and quantum-dot size as a function of annealing time at 100°C.

**Figure 7:**
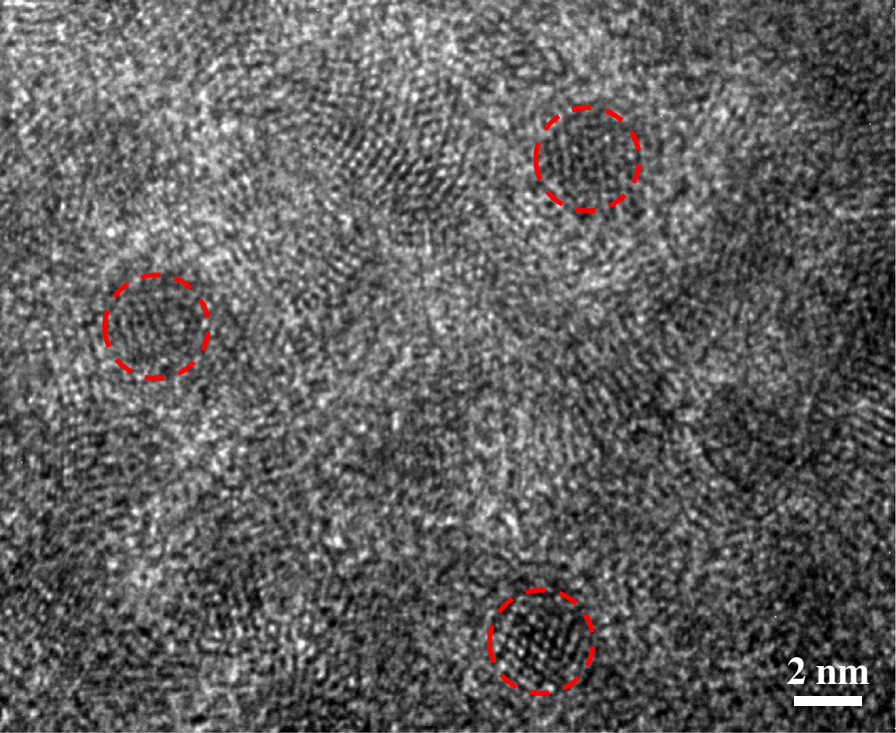
TEM image of the 12-h-annealed CdS quantum dots at 100°C (approximately 3.7 nm in diameter).

The overall properties of CdS nanoparticles were summarized in Figure 6. It is clear that the emission decay for the bare CdS is dominated by nonradiative decay, which is mediated by high-density trap states in the nanoparticles. However, the facile annealing reduces the local strain and nonradiative recombination center and thereby exhibits longer carrier lifetime and higher quantum efficiency, consistent with the TEM data of Figure 7. In this way, we straightforwardly synthesized highly luminescent CdS nanoparticles, from 1% to 29% in quantum efficiency.

## Conclusions

The luminescence properties of CdS quantum dots in the initial growth stage were examined in connection with a simple annealing process. Both the accumulated defect states and nonradiative recombination rates were reduced, and these correlations were confirmed systematically by diffraction, absorption, and time-resolved photoluminescence. Consequently, the highly luminescent (quantum efficiency of 29% from the initial 1%) and defect-free nanoparticles were obtained by a facile annealing process.

## Competing interests

The authors declare that they have no competing interests.

## Authors’ contributions

JIK and JK drafted and revised the manuscript. JL carried out the synthetic experiments and characterizations. DRJ, HK, HC, SL, SB, and SK participated in the scientific flow. BP conceived of the study and participated in its design and coordination. All authors read and approved the final manuscript.
